# Supplementary material for: Direct observation of tRNA-chaperoned folding of a dynamic mRNA ensemble
Source: Nat Commun. 2023 Sep 6;14:5438. doi: 10.1038/s41467-023-41155-3 (PMC10482949; doi:10.1038/s41467-023-41155-3)
Supplement: Supplementary file 1 — Supplementary Information [file 41467_2023_41155_MOESM1_ESM.pdf]

Supplementary Materials for:

Direct observation of tRNA-chaperoned folding of a dynamic mRNA ensemble

Krishna C. Suddala, Janghyun Yoo, Lixin Fan, Xiaobing Zuo, Yun-Xing Wang, Hoi Sung Chung, & Jinwei Zhang

Supplementary Tables 1-2
Supplementary Figures 1-10

U linker length	K_d (μM)	log K_a	ΔH (kcal mol^{-1})	-TΔS (kcal mol^{-1})
0*	29 ± 3*	4.5*	-25*	19*
1	0.96 ± 0.03	6.02	-20.2	12.1
2	0.61± 0.04	6.21	-21.9	13.6
3	0.51 ± 0.04	6.29	-25.2	16.7
4	0.72 ± 0.07	6.14	-25.4	17.1
5	1.47± 0.38	5.84	-25.2	17.4
10	3.29 ± 0.19	5.48	-24.4	18.1

Supplementary Table 1. ITC-derived thermodynamic parameters of tRNA binding to ΔΨ T-boxes harboring different lengths of U-linkers. Data shown are mean ± S.D. from two or three biologically independent replicates (n). n = 2 for U-linkers 3, 4, and 5, n = 3 for U-linkers 1 and 10. *data from Suddala et al.,[1] under identical conditions. These thermodynamic parameters were subject to larger errors due to very low affinity and less sigmoidal shapes of the thermograms.

Sample	τ_1 (ns)	A1	τ_2 (ns)	A2	τ_3 (ns)	A3	τ_{avg} (ns)	χ^2
A16 Stem I	0.47	0.52	2.35	0.25	6.69	0.23	2.39±0.02	1.04
A16 Apo	0.52	0.48	2.37	0.24	6.70	0.28	2.69±0.02	0.98
A16 Complex	0.55	0.45	2.23	0.45	5.81	0.10	1.83±0.04	1.26
A19 Stem I	0.29	0.75	2.01	0.11	7.79	0.14	1.51±0.01	1.04
A19 Apo	0.34	0.74	1.89	0.11	8.01	0.15	1.63±0.05	1.05
A19 Complex	0.22	0.90	1.76	0.05	7.87	0.05	0.66±0.02	1.19

Supplementary Table 2. 2AP fluorescence lifetimes and amplitudes. Individual lifetimes (τ_1-τ_3), amplitudes (A1 – A3) and amplitude-weighted mean lifetime (τ_{avg}) values determined from 3-exponential fits to 2AP fluorescence decays. Apo: T-box stem I-II-IIA/B construct. Mean lifetime (τ_{avg}) values shown are mean ± S.D. from three biologically independent samples.

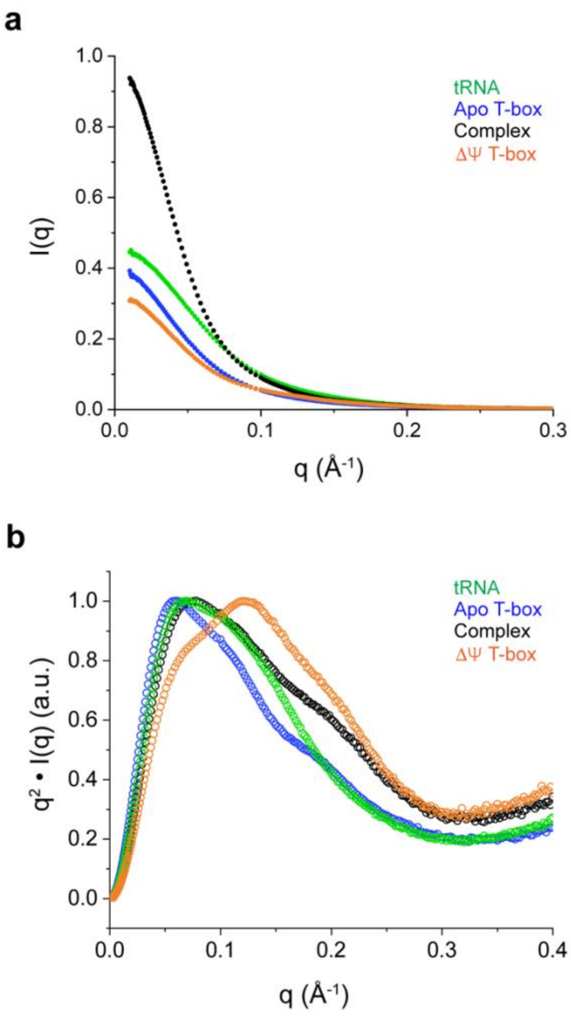

Supplementary Figure 1: **SAXS analyses of *N. farcinica ileS* T-box RNAs, tRNA^{Ile}, and their complex.**

a Plots of SAXS experimental scattering intensities (I(q)) of tRNA^{Ile} (green), Apo T-box (blue, nts 1-98), their complex (black), and the free ΔΨ T-box (orange, nts 1-77) RNAs. **b** Kratky plots of the data in (**a**), showing that these RNA samples are well folded. Source data are provided as a Source Data file.

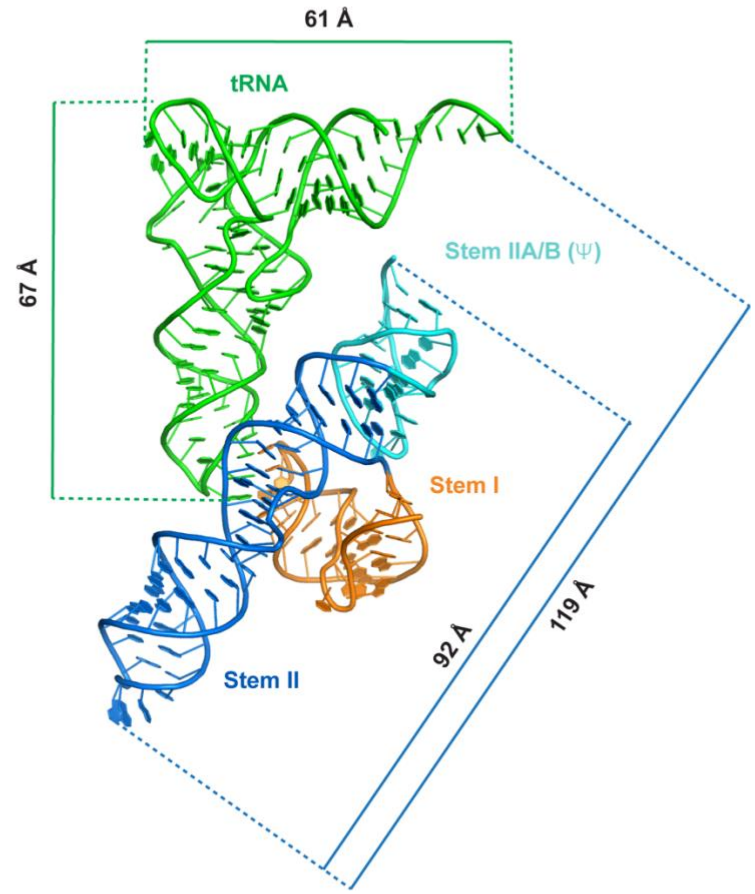

Supplementary Figure 2. Dimensions and measurements of the *N. farcinica ileS* T-box-tRNA co-crystal structure.
Maximum inter-atomic distances estimated from the crystal structure (PDB ID: 6UFM)[1] are indicated and compared with maximum particle dimensions (D_{max}) derived from SAXS pairwise distance distribution functions (PDDF, **Fig. 2c**).

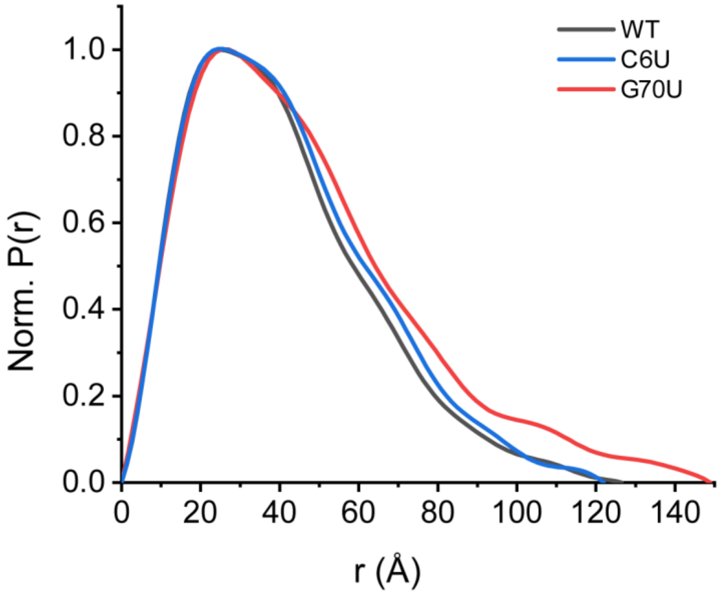

Supplementary Figure 3. SAXS analyses of WT and variant T-box RNAs.
Pair-wise distance distribution function (PDDF) plots for WT (black), C6U (stem I K-turn region, blue), and G70U (stem II S-turn region, red) T-box RNAs. Source data are provided as a Source Data file.

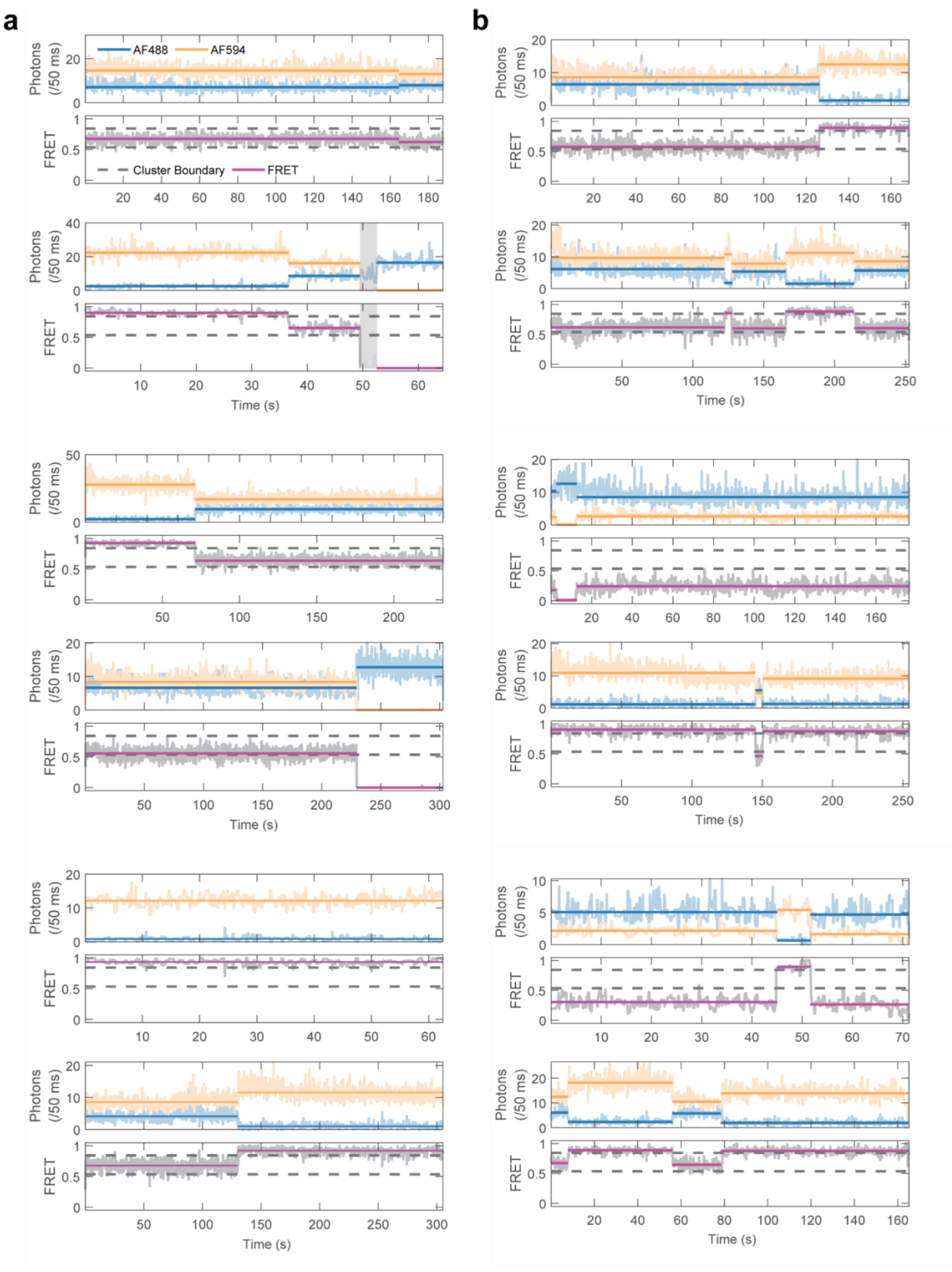

a
b
AF488
AF594
Photons (/50 ms)
FRET
Cluster Boundary
FRET
Time (s)

Supplementary Figure 4. Additional representative two-color smFRET trajectories.
a, b Two-color binned (50 ms bin time) trajectories of donor (AF488, blue) and acceptor (AF594, orange) fluorescence and FRET efficiencies (gray/purple) for the T-box RNA in the absence (**a**) or presence of 50 nM tRNA (**b**). Background, donor leak, γ-factors, and direct excitation of acceptors are corrected.

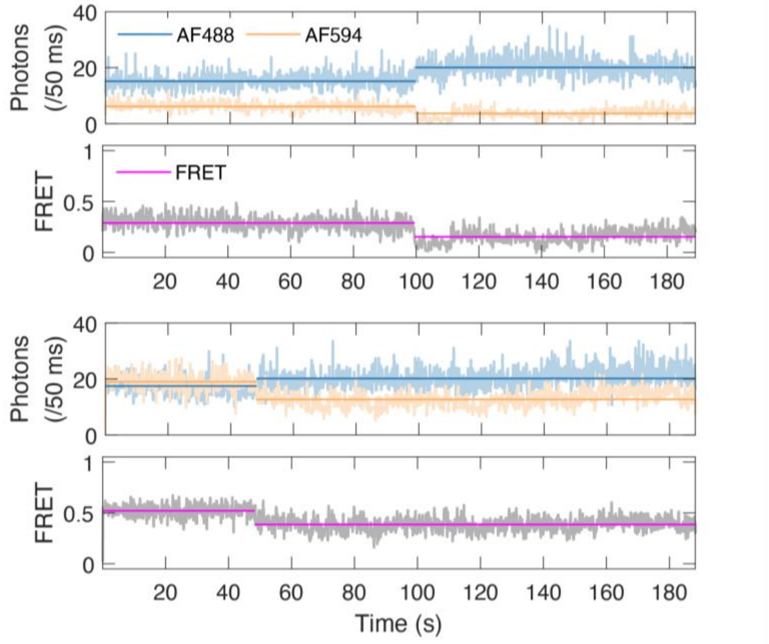

Supplementary Figure 5: **two-color smFRET trajectories of T-box RNA in 1 mM of Mg^{2+}.** Two-color binned (50 ms bin time) trajectories of donor (AF488, blue) and acceptor (AF594, orange) fluorescence and FRET efficiencies (gray/purple) for the T-box RNA in the absence of tRNA.

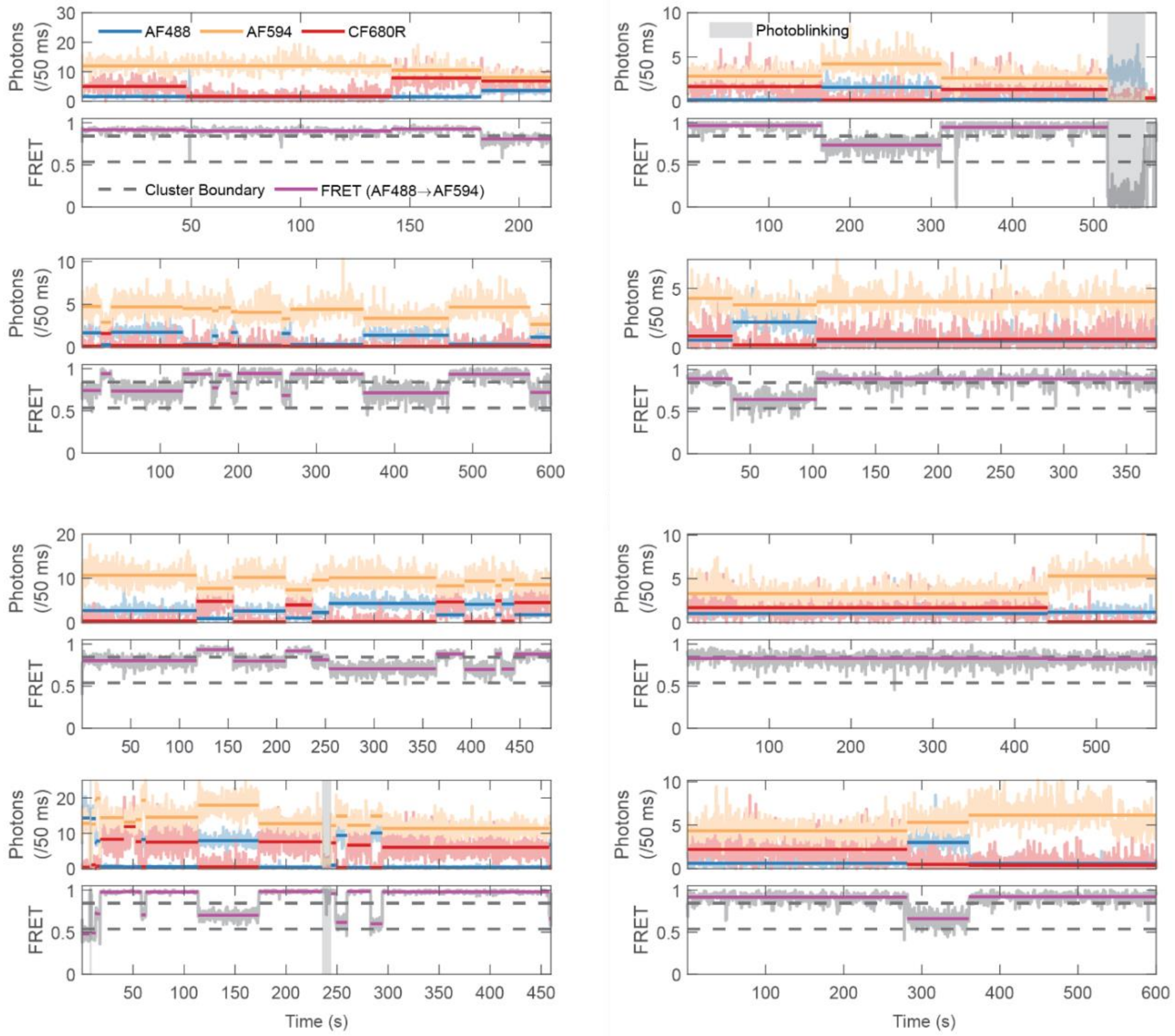

Supplementary Figure 6: **Additional representative three-color smFRET trajectories**. Three-color binned (50 ms bin time) trajectories of donor (AF488, blue), acceptor 1 (AF594, orange) and acceptor 2 (CF680R, red) fluorescence and pseudo two-color FRET efficiencies (gray/purple) for the T-box RNA in the absence of tRNA-ASL. Background, leak, γ-factors, and direct excitation of acceptors are corrected.

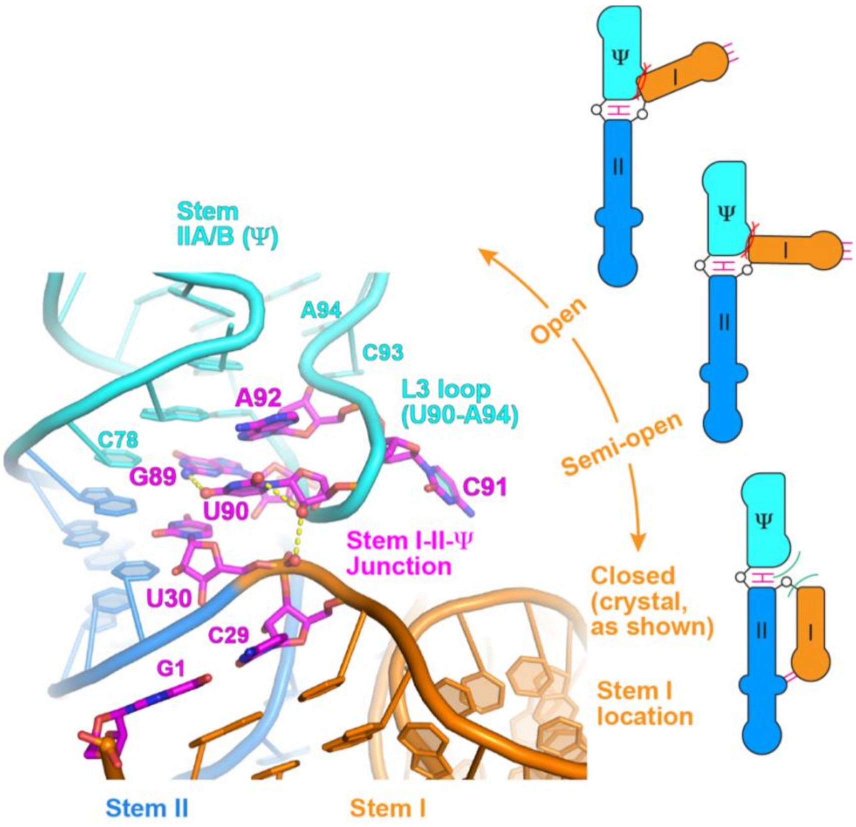

Supplementary Figure 7: **Local structure of the Stem I-II-Ψ junction**
Key residues located at the Stem I-II-Ψ junction (magenta) as observed in the co-crystal structure (PDB ID: 6UFM)[1]. Hydrogen bonds are shown as yellow dashes. In the closed form of the T-box (U3, B3 states, bottom cartoon), the junction appears relaxed and free of steric clashes (green arcs). When the T-box opens up (middle and upper cartoons), the base of stem I is projected to be closer to the pseudoknot, especially the L3 loop region (U90-A94). This juxtaposition and proximity may alter or restrain the local conformation of the junction (red arcs), thereby slowing the interconversion between different conformational states of the junction. This local structural difference at the junction may persist through tRNA binding episodes and produce the observed conformational memory effect.

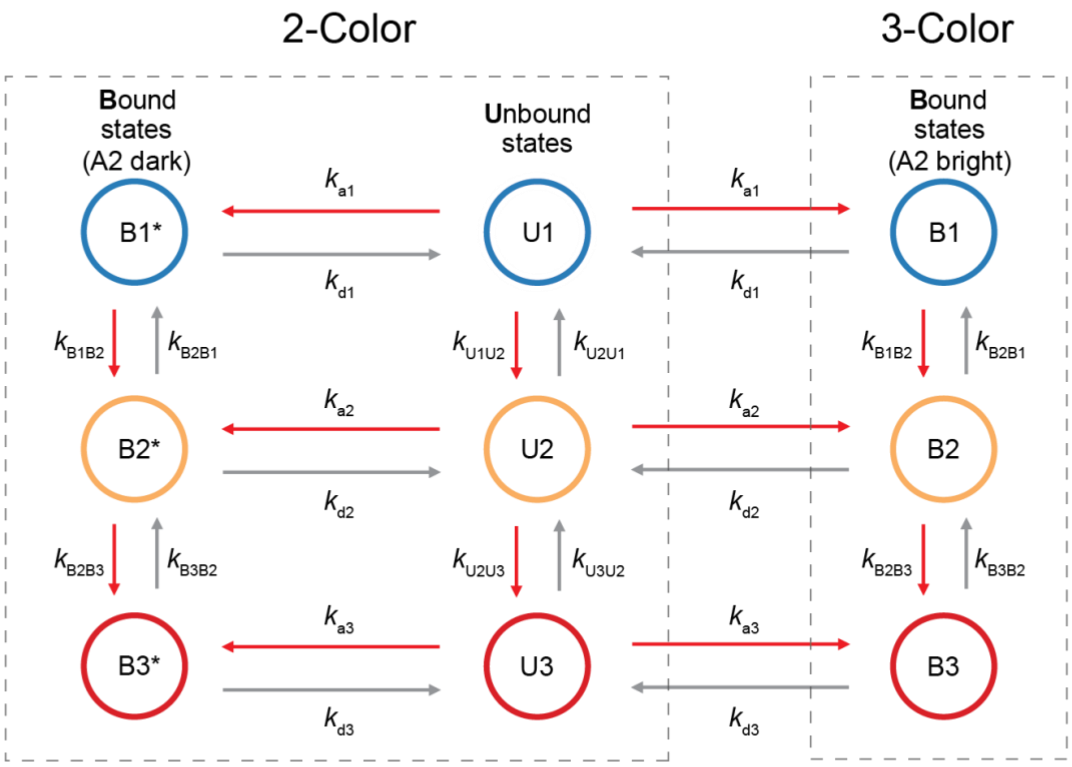

Supplementary Figure 8. **Detailed kinetic model of tRNA binding to the T-box ensemble.** The 9-state kinetic model includes three tRNA-unbound states (U1, U2 and U3, 2-Color), three tRNA-bound states with an active acceptor 2, termed A2 (B1, B2 and B3, 3-Color), and three A2-dark bound state (B1*, B2* and B3*, 2-Color, due to incomplete labeling, photobleaching or otherwise inactive acceptor 2) with the same binding and interconversion kinetic parameters as those of the A2-bright bound states. Numbers 1-3 denote open, semi-open, and closed conformations, respectively. Letters B and U denote tRNA-bound and unbound states, respectively.

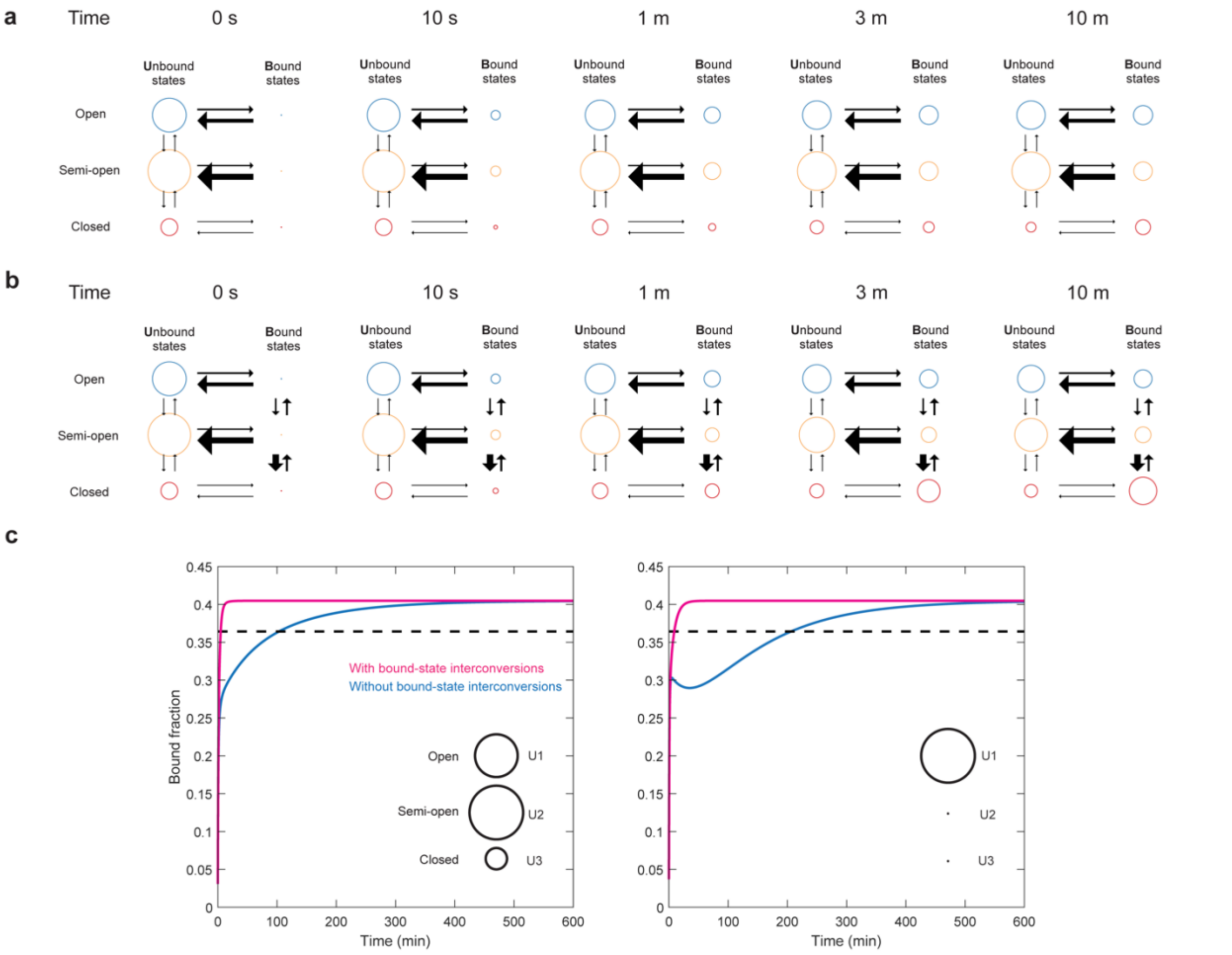

Supplementary Figure 9. **Effects of bound-state interconversions and pre-binding conformational equilibration on tRNA-binding kinetics.**
a, b Simulations of the populations of each physical state as a function of time in the absence (**a**) or presence (**b**) of bound-state interconversions (vertical arrows), starting from pre-equilibrated unbound states. Colored and styled as in Fig. 4c. The areas of the circles are proportional to the population of each state and the thickness of arrows is proportional to the rate. **c** Plots of overall tRNA-bound populations as a function of time in the absence (blue) or presence (magenta) of bound-state interconversions, starting from pre-equilibrated unbound states (left panel) or from unequilibrated unbound states 1, i.e.100 % open conformation, U1 (right panel). The dashed lines denote 90 % of the equilibrium bound population. Source data are provided as a Source Data file.

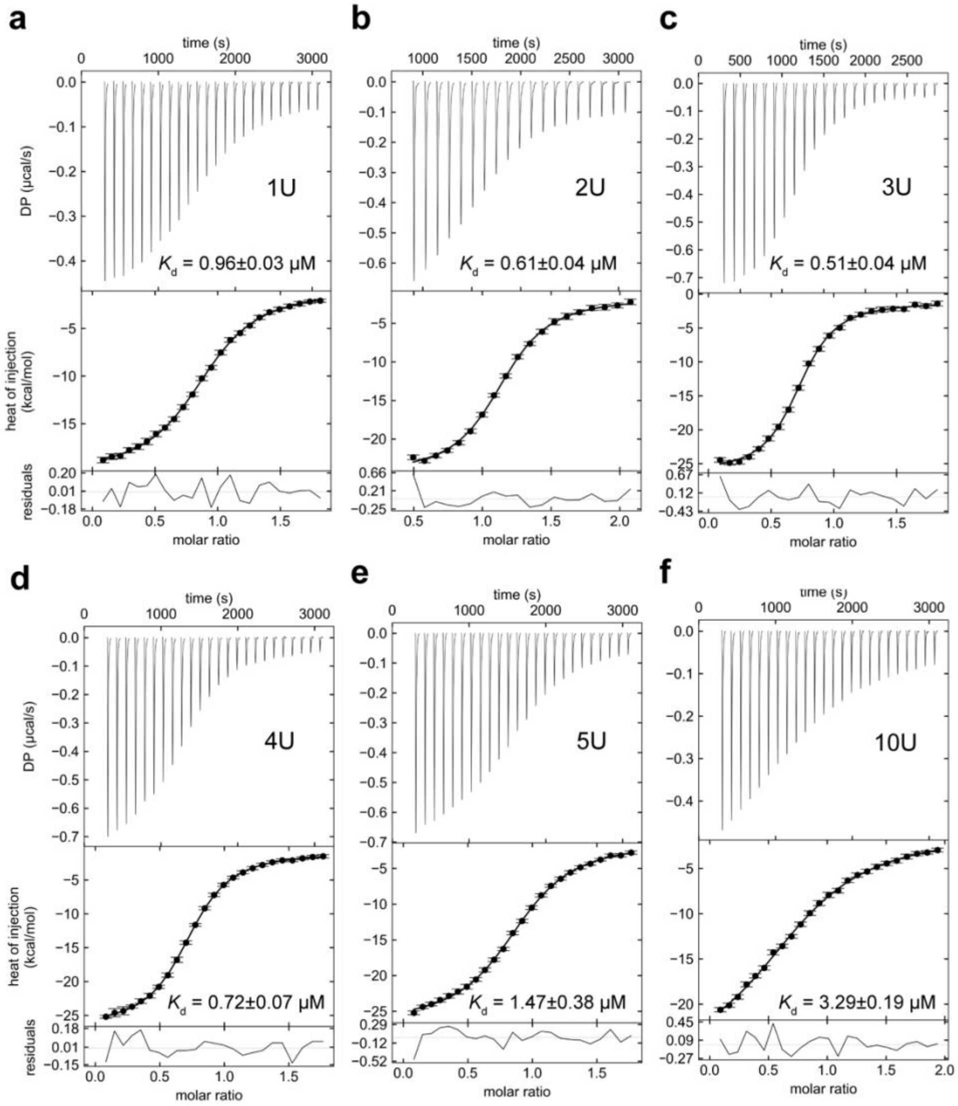

Supplementary Figure 10. Representative ITC thermograms for tRNA binding to ΔΨ T-boxes harboring polyuridine linkers.

a-f ITC thermograms for $tRNA^{Ile}$ binding to ΔΨ T-box RNAs with different polyuridine linker lengths:1U (**a**), 2U (**b**), 3U (**c**), 4U (**d**), 5U (**e**) and 10U (**f**). The K_d values are indicated for each T-box variant, and are mean ± S.D. from two or three biologically independent replicates (N). N = 2 for U-linkers 3, 4, and 5; N = 3 for U-linkers 1 and 10. Thermodynamic parameters are summarized in Supplementary Table 1.

Supplementary References.

1. Suddala, K.C. & Zhang, J. High-affinity recognition of specific tRNAs by an mRNA anticodon-binding groove. *Nat Struct Mol Biol* **26**, 1114-1122 (2019).
