# Supplementary material for: Direct observation of tRNA-chaperoned folding of a dynamic mRNA ensemble
Source: Nat Commun. 2023 Sep 6;14:5438. doi: 10.1038/s41467-023-41155-3 (PMC10482949; doi:10.1038/s41467-023-41155-3)
Supplement: Supplementary file 3 — Reporting Summary [file 41467_2023_41155_MOESM3_ESM.pdf]

nature portfolio

Corresponding author(s): Hoi Sung Chung; Jinwei Zhang

Last updated by author(s): Jul 28, 2023

Reporting Summary

Nature Portfolio wishes to improve the reproducibility of the work that we publish. This form provides structure for consistency and transparency in reporting. For further information on Nature Portfolio policies, see our Editorial Policies and the Editorial Policy Checklist.

Statistics

For all statistical analyses, confirm that the following items are present in the figure legend, table legend, main text, or Methods section.

n/a	Confirmed	
☐	☒	The exact sample size (*n*) for each experimental group/condition, given as a discrete number and unit of measurement
☐	☒	A statement on whether measurements were taken from distinct samples or whether the same sample was measured repeatedly
☒	☐	The statistical test(s) used AND whether they are one- or two-sided<br>*Only common tests should be described solely by name; describe more complex techniques in the Methods section.*
☒	☐	A description of all covariates tested
☒	☐	A description of any assumptions or corrections, such as tests of normality and adjustment for multiple comparisons
☐	☒	A full description of the statistical parameters including central tendency (e.g. means) or other basic estimates (e.g. regression coefficient) AND variation (e.g. standard deviation) or associated estimates of uncertainty (e.g. confidence intervals)
☒	☐	For null hypothesis testing, the test statistic (e.g. *F*, *t*, *r*) with confidence intervals, effect sizes, degrees of freedom and *P* value noted<br>*Give P values as exact values whenever suitable.*
☒	☐	For Bayesian analysis, information on the choice of priors and Markov chain Monte Carlo settings
☒	☐	For hierarchical and complex designs, identification of the appropriate level for tests and full reporting of outcomes
☒	☐	Estimates of effect sizes (e.g. Cohen's *d*, Pearson's *r*), indicating how they were calculated

Our web collection on statistics for biologists contains articles on many of the points above.

Software and code

Policy information about availability of computer code

Data collection	Symphotime v5 (Picoquant), Matlab 2019b, Datastation (Horiba).
Data analysis	Matlab. Binding measurements were plotted and analyzed in OriginPro Version 2018. SAXS data were analyzed using ATSAS suite version 3. Ensemble 2AP lifetime measurements were analyzed using Horiba DAS6.

For manuscripts utilizing custom algorithms or software that are central to the research but not yet described in published literature, software must be made available to editors and reviewers. We strongly encourage code deposition in a community repository (e.g. GitHub). See the Nature Portfolio guidelines for submitting code & software for further information.

Data

Policy information about availability of data

All manuscripts must include a data availability statement. This statement should provide the following information, where applicable:

- Accession codes, unique identifiers, or web links for publicly available datasets
- A description of any restrictions on data availability
- For clinical datasets or third party data, please ensure that the statement adheres to our policy

All data supporting the main conclusion are included in the manuscript and associated Source Data. The study used PDB ID: 6UFM (https://doi.org/10.2210/pdb6UFM/pdb).

Human research participants

Policy information about studies involving human research participants and Sex and Gender in Research.

Reporting on sex and gender	N/A
Population characteristics	N/A
Recruitment	N/A
Ethics oversight	N/A

Note that full information on the approval of the study protocol must also be provided in the manuscript.

Field-specific reporting

Please select the one below that is the best fit for your research. If you are not sure, read the appropriate sections before making your selection.

☒ Life sciences ☐ Behavioural & social sciences ☐ Ecological, evolutionary & environmental sciences

For a reference copy of the document with all sections, see nature.com/documents/nr-reporting-summary-flat.pdf

Life sciences study design

All studies must disclose on these points even when the disclosure is negative.

Sample size	Biochemical and Biophysical assays were performed on independent biological duplicates or triplicates in vitro, and indicated in the Methods, and legends to Supplementary Tables 1-2 and supplementary Fig. 10. Number of single molecules analyzed are indicated in Fig. 3c,3e, 5d. Single-molecule data follow Poisson statistics. Then, the error (SD) is square root of the sample size. So, if the sample size is larger than 100, the relative error becomes smaller than 10%. So, the measurement from the sample size of several hundreds to 1000 in this study will be sufficiently accurate.
Data exclusions	We observe the spectral shift of the donor fluorophore (Alexa 488) occasionally in single molecule trajectories, which changes the FRET efficiencies. Therefore, the trajectories affected by the spectral shift were excluded from the analysis. The spectral shift can be identified by increased donor leak into the acceptor channel as described in several papers including Chung et al. PNAS vol. 106, 11837 (2009). Impurity molecules were also identified by irregular fluorescence lifetimes and excluded from the analyses.
Replication	All independent biological replications were successful and included. Exact numbers are included in figure legends.
Randomization	No experimental groups were present in this study. There was no allocation. Randomization is not relevant in this study.
Blinding	No group allocation in this study. Individual RNA molecules have no identifiers or groups. Blinding is not relevant for the study.

Reporting for specific materials, systems and methods

We require information from authors about some types of materials, experimental systems and methods used in many studies. Here, indicate whether each material, system or method listed is relevant to your study. If you are not sure if a list item applies to your research, read the appropriate section before selecting a response.

Materials & experimental systems

n/a	Involved in the study	
☒	☐	Antibodies
☒	☐	Eukaryotic cell lines
☒	☐	Palaeontology and archaeology
☒	☐	Animals and other organisms
☒	☐	Clinical data
☒	☐	Dual use research of concern

Methods

n/a	Involved in the study	
☒	☐	ChIP-seq
☒	☐	Flow cytometry
☒	☐	MRI-based neuroimaging
